# Delayed Presentation of Iliac Vein Injury: A Severe Complication at Retropubic Midurethral Sling Arm Removal

**DOI:** 10.1155/2019/4732356

**Published:** 2019-04-16

**Authors:** Pansy Uberoi, Forrest Jellison

**Affiliations:** San Antonio Uniformed Services Health Education Consortium, USA

## Abstract

Midurethral slings are the most common treatment for female stress urinary incontinence. Perioperative vascular injuries during placement of a retropubic midurethral sling (RMUS) are uncommon but have been described. The objective of this case report is to describe a complication of delayed presentation from a vascular injury at the time of retropubic sling arm removal not previously documented in the literature. This life-threating complication should be considered and precautions should be taken at retropubic sling arm removal. Prevention is accomplished by proper visualization of pelvic vasculature and/or eliminating tension on sling before excision.

## 1. Introduction

Retropubic midurethral mesh slings (RMUS) are a standard treatment option for the management of stress urinary incontinence. The recent American Urologic Association guideline for surgical management of female stress urinary incontinence (SUI) described MUS as the most widely studied with follow-up data over more than 15 years [1]. Despite its well documented safety and efficacy, the RMUS is associated with complications. Common complications can be divided into intraoperative and postoperative issues, and the management and report outcomes are well described [2, 3]. Intraoperative hemorrhage has been described with significant vessel injury found less than 0.7% and hematoma has been described in approximately 2% of patients [4]. Delayed and asymptomatic vascular injuries have not been reported.

In this case report, we describe the asymptomatic delayed presentation of a RMUS located in the wall of the external iliac vein that leads to catastrophic bleeding requiring emergent repair at the time of sling arm removal.

## 2. Case Presentation

A 69-year-old female underwent placement of RMUS in May 2014 for SUI by a surgeon from another institution. She developed de novo left groin/inner thigh pain, vaginal pain, and abdominal pain at the site of left sling arm and de novo overactive bladder and dysfunctional voiding. After follow-up and discussion with her original surgeon, they decided to proceed with a sling incision six months from her sling placement. After the sling revision, her pain and urinary symptoms did not improve, and she was self-referred to our institution for evaluation.

After a thorough evaluation that included examination, cystoscopy, labs, CT scan, and Urodynamics (UDS) that revealed pertinent findings of trigger point tenderness at the left suprapubic trocar incision site and vaginally in the left levator muscles, the left trocar incision site was unusually more superior and lateral than is typically found on examination, and UDS findings demonstrated urodynamic stress incontinence and bladder outflow obstruction. After extensive counseling, patient underwent transvaginal and suprapubic removal of the remaining left retropubic arm and remaining suburethral portion of the sling.

## 3. Interventions/Surgical Course

Approximately one year after her RMUS, she was taken to the operating room with the plan for excision of the remaining suburethral portion of the mesh sling and partial removal of the left retropubic arm. We made an inverted U-incision and then identified the suburethral portion of the mesh sling. During her suburethral revision, the right side of her sling was partially excised without entering the retropubic space, and we noted that the remaining left portion of the mesh sling was nearly penetrating the urethral mucosa. This portion of the sling was nearly penetrating the mucosal layer, located submucosally with extensive fibrosis that required a small urethrotomy to excise it completely. The urethrotomy was closed primarily with absorbable suture and left a martius flap was interposed to provide tissue coverage.

Attention was then taken to the removal of the left retropubic arm of the sling. The incision scar from the trocar of the remaining left portion of the sling was identified to be 5 cm superior and 6 cm lateral to the pubic symphysis, and, during vaginal exploration, the sling was noted to enter the obturator internus and iliococcygeus muscles. Given the location of the trocar site, preoperatively we discussed partial removal of the left arm of the mesh to the level of the abdominal fascia without removal of RMUS retropubic arms as they are potentially located adjacent to the iliac vessels. We removed the suburethral sling from a vaginal approach to where it entered the retropubic space, and the sling was embedded into the periosteum and then we began the removal of the sling abdominally to the level of the rectus fascia. A 3 cm mini-Gibson incision was used in the left lower abdomen over the scar from initial sling placement to allow for adequate exposure and reduce morbidity of a larger incision. The sling was dissected free to the level of the abdominal fascia. Careful attention was paid not to injure sounding structures, and the sling was pulled on traction superiorly and excised under direct visualization.

Immediately after excision of the abdominal arm of the sling, approximately 200 ml of blood loss was experienced. Bleeding was controlled with direct pressure to the area. Expeditiously, the abdominal incision was extended from a mini-Gibson to a full Gibson to allow visualization of bleeding. After obtaining exposure, the source of bleeding was identified with the sling embedded in the wall of the vein creating a traction injury in the medial wall of the left external iliac vein. The remaining visible sling was excised from the external iliac vein, and the venotomy was repaired with primary closure. Intraoperatively Vascular Surgery team was consulted and examined the repaired vein and determined that lumen diameter was adequate and assessed for any thrombus. She was taken to the ICU postoperatively where, after extubation, she complained of worsened left lower extremity pain which, at that time, also appeared to have some purple discoloration in her left foot. Her symptoms were consistent with acute venous congestion from a left external vein thrombus that was diagnosed on CT arteriogram and venogram. The patient returned to the operative room with the Vascular Surgery team. A small remaining intraluminal portion of mesh was identified and removed from the sling in the external iliac vein and was associated with the thrombus. Venotomy, thrombectomy, and excision of mesh were performed, and the vein was subsequently repaired with a contralateral saphenous vein patch.

## 4. Aftercare

After recovery from her surgery and completion of her course of anticoagulation she underwent placement of an autologous rectus fascia pubovaginal sling for her persistent SUI. She noted significant improvement in her SUI and since the prior surgery reported complete resolution of vaginal and leg pain, with 70% improvement in abdominal pain, symptoms at 18-month follow-up.

## 5. Discussion

Despite its reported safety and efficacy for treatment of female stress urinary incontinence, placement of retropubic midurethral mesh sling has known complications. Management and outcomes of these complications are well described in the literature [2, 3]. Vascular injury with MUS placement is rare, and most are identified intraoperatively as active extravasation of blood or hemodynamic instability. However, this case represents delayed recognition of vascular injury at the time of sling removal. It was unexpected to find that the sling is transverse through the wall of the external iliac vein, due to there being no reported vascular complications at sling placement or any vascular complications postoperatively.

In our literature search, one case of delayed diagnosis of external iliac vein injury was noted. However, the patient described in that report sustained several complications at index surgery and presented with back and abdominal bloating and gross hematuria with sling perforation and bladder stones. At the time of reoperation, the surgeon had decided to perform a laparotomy and remove the entire retropubic sling as per her request, and intraoperatively it was noted that the sling penetrated into bladder lumen, created scar tissue surrounding the obturator nerve, and was intraluminal in the contralateral external iliac vein [5].

Although the overall frequency of vascular injury during MUS placement is rare, a comprehensive knowledge of pelvic anatomy is necessary to prevent injury [6]. Sufficient surgical experience and training are paramount to the prevention of morbidity and increased safety. The AUA/SUFU position statement is that surgeons are to be trained rigorously in the pelvic anatomy and in recognition of complications [7]. In this case, the patient reported that the outside surgeon had stated that he had minimal experience performing retropubic midurethral slings.

**Figure 1:**
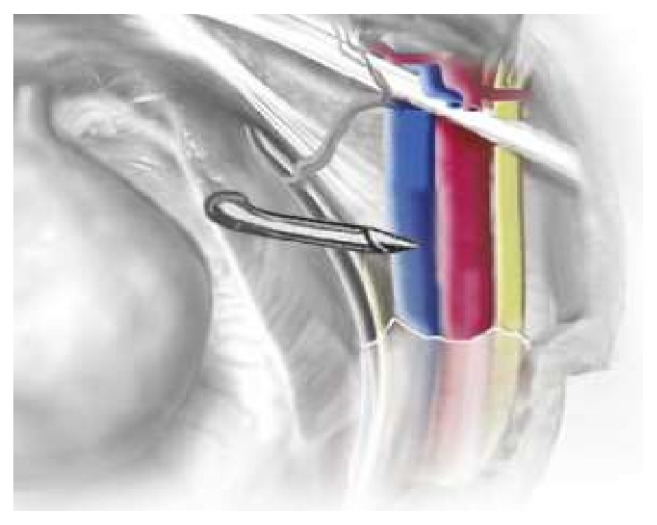
Trocar position relative to external iliac vein at placement.

In our case, the patient experienced a life-threatening complication during the removal of the retropubic arms of the sling. A venotomy resulted from traction of the sling on the external iliac vein tension prior to excision. We suspect that the RMUS was into but not through the intimal layer of the vessel during placement (Figure 1). Given the possible proximity of sling arms to pelvic vessels, open/laparoscopic laparotomy should be considered by the operating surgeon when removing suprapubic sling arms to prevent vascular injury and to identify such injuries if they occur. Although still investigational, there is possibly a role for pelvic/translabial ultrasound in perioperative planning [8, 9]. Alternately, if we had placed minimal tension when pulling on the sling before excising it, this would have resulted in no vascular injury. Additionally, this would have resulted in using the planned 3 cm incision and would have decreased the morbidity due to the incision size.

In conclusion, prevention of vascular injury is vital when removing the retropubic sling arms even when the patient has no prior vascular complications at sling placement. This is accomplished by proper visualization of pelvic vasculature and/or minimizing tension on sling during excision.
